# Therapeutic JAK inhibition does not impact lung injury during viral or bacterial pneumonia in male mice

**DOI:** 10.14814/phy2.70232

**Published:** 2025-02-07

**Authors:** Lokesh Sharma, Ravineel B. Singh, Caden Ngeow, Rick van der Geest, Alexis M. Duray, Nathanial J. Tolman, Bryan J. McVerry, Charles S. Dela Cruz, John F. Alcorn, William Bain, Keven M. Robinson

**Affiliations:** ^1^ Division of Pulmonary, Allergy, Critical Care and Sleep Medicine, Department of Medicine University of Pittsburgh Pittsburgh Pennsylvania USA; ^2^ Division of Pulmonary Medicine, Department of Pediatrics University of Pittsburgh Pittsburgh Pennsylvania USA

**Keywords:** bacteria super‐infection, influenza, JAK inhibition, methicillin‐resistant staph aureus

## Abstract

Influenza infections are often complicated by secondary bacterial infections such as MRSA pneumonia, which increase morbidity and mortality. Viral infections lead to an inflammatory response that includes elevated levels of IL‐6 and interferons. IL‐6 activates the JAK/STAT signaling pathway, amplifying downstream inflammation. Given the clinical efficacy of the JAK inhibitor baricitinib in reducing disease severity in COVID‐19, we evaluated its impact in a murine model of influenza, MRSA, and post‐influenza MRSA pneumonia. Additionally, because IL‐6 inhibitory therapies have improved outcomes during COVID‐19, we evaluated the impact of IL‐6 deletion on post‐influenza MRSA pneumonia. In our studies, baricitinib effectively inhibited the JAK/STAT pathway in the lungs, as demonstrated by decreased interferon‐stimulated genes (ISGs) and STAT3 phosphorylation. Despite this inhibition, baricitinib did not cause a global suppression of cytokines. Notably, baricitinib treatment did not impair either antiviral or antibacterial host immunity, inflammatory cell recruitment, or lung tissue injury. IL‐6 deficiency did not alter weight loss, inflammatory cell recruitment, or bacterial burden during post‐influenza MRSA pneumonia. These findings suggest that both JAK inhibition via baricitinib and IL‐6 deletion do not enhance host defense or limit tissue injury in murine models of influenza and post‐influenza MRSA pneumonia.

## 1 INTRODUCTION

Novel influenza A viruses can emerge and result in pandemics, leading to global disruption and significant morbidity and mortality. Even in the absence of pandemics, seasonal influenza infection causes significant health burden, resulting in 290,000–650,000 deaths worldwide (Tyrrell et al., 2021). An exacerbated inflammatory response during influenza infection, both locally and systemically, is thought to contribute to disease severity (Gu et al., 2021). Lung‐specific pathological manifestations include the accumulation of both innate and adaptive immune cells in the lung parenchyma and alveolar spaces. Moreover, these pathological changes can persist even after viral clearance, which suggests that immunomodulatory therapies may be beneficial (Keeler et al., 2018). IL‐6 levels are elevated in viral infections, including both influenza and SARS‐CoV‐2 (Dienz et al., 2012; Wang et al., 2022). IL‐6 activates the JAK/STAT (janus kinase—signal transducer and activator of transcription) signaling pathway to initiate gene transcription of cytokines and chemokines critical to inflammatory and acute phase responses. Several immunomodulatory therapies targeting IL‐6 and JAK signaling have demonstrated beneficial effects during SARS‐CoV‐2 infection, leading to decreased disease severity and mortality. Among these, the JAK inhibitor baricitinib showed significant clinical benefits in severe COVID‐19 patients, with or without antiviral therapies (Kalil et al., 2021; Marconi et al., 2021). Baricitinib reduced mortality, time to recovery, and adverse events during SARS‐CoV‐2 infection (Ely et al., 2022; Group RC, 2022; Marconi et al., 2021; Wolfe et al., 2022). Similarly, inhibition of interleukin 6 (IL‐6) signaling using monoclonal antibodies was associated with a decreased inflammatory response and overall improvement in clinical outcomes (REMAP‐CAP Investigators et al., 2021; Salama et al., 2021). IL‐6 receptor antagonists reduced mortality and organ support‐free days during SARS‐CoV‐2 infection (Group RC, 2021; REMAP‐CAP Investigators et al., 2021) (Group WHOREAfC‐TW et al., 2021). Based on this clinical experience, others have proposed that these agents may also be of use during influenza infection (Hays et al., 2022). Although SARS‐CoV‐2 infection and influenza infection share some immunologic response features, there are important differences in host susceptibility against the two viruses and additional investigation can help determine if therapeutic treatments that are beneficial during SARS‐CoV‐2 may also be beneficial during influenza infection.

Severe influenza infection is often complicated by bacterial super‐infection and bacterial pneumonia increases influenza‐associated morbidity and mortality (Morens et al., 2008). During the most recent influenza pandemic in 2009, bacterial pneumonia complicated 25%–50% of severe infections in both children and adults (Gill et al., 2010; Martin‐Loeches et al., 2011; Randolph et al., 2011; Rice et al., 2012; Shieh et al., 2010). *Staphylococcus aureus* and *Streptococcus pneumoniae* are two prominent organisms that cause secondary bacterial pneumonia (Blyth et al., 2013; Gill et al., 2010; Martin‐Loeches et al., 2011; Randolph et al., 2011; Rice et al., 2012; Shieh et al., 2010). Therefore, we sought to determine how inhibition of IL‐6 and JAK signaling affects both influenza infection and post‐influenza MRSA pneumonia. For JAK inhibition, we used the FDA‐approved drug baricitinib, given its proven effectiveness in mouse models (Gu et al., 2023; Matsushita et al., 2023). Mice received daily baricitinib starting on Day 3 post‐influenza in order to mimic human illness where patients typically present to healthcare settings a few days after the onset of symptoms and to provide a translational model of JAK inhibition during influenza infection. Due to the ineffectiveness of IL‐6 modulatory therapies such as tocilizumab (Lokau, Kleinegger, et al., 2020; Lokau, Waetzig, et al., 2020; Okazaki et al., 2002) in mouse models, we also used IL‐6‐deficient mice to model IL‐6 inhibition during respiratory infection. Our overall goal was to determine if targeting JAK or IL‐6 signaling provides a benefit during viral or viral/bacterial infection in a preclinical animal model.

## 2 METHODS

### 2.1 Animal studies

Nine‐ to ten‐week‐old male wildtype (WT) C57BL/6 mice were purchased from Taconic Farms. Six‐ to eight‐week‐old male B6.129S2‐*Il6* ^*tm1Kopf*^/J (IL‐6 KO) mice and WT controls were purchased from The Jackson Laboratory. Mice were maintained under specific pathogen‐free conditions at the University of Pittsburgh. All the studies used age‐ and sex‐matched mice. In specific experiments, mice were treated as indicated with baricitinib (AdooQ Bioscience, Irvine, CA). A 10 mg/kg dose of baricitinib (Adooq BioScience Catalog # LY3009104) was selected based on prior studies (Gu et al., 2023; Matsushita et al., 2023; Tuttle et al., 2020). Baricitinib was suspended in 0.5% methylcellulose (Sigma Aldrich) and administered by oral gavage daily on Days 3–6 post‐influenza infection. Control animals received 0.5% methylcellulose by oral gavage as a vehicle control on Days 3–6 post‐influenza infection. Mice were euthanized with pentobarbital at a dose of 300 mg/kg administered via intraperitoneal injection.

### 2.2 Bacterial and viral infections

MRSA (USA 300) was cultured as detailed by ATCC instructions in casein hydrolysate yeast extract–containing modified medium for 18 h to stationary growth phase. Mice were inoculated with 5 × 10^7^–1 × 10^8^ CFU of MRSA in 50 μL sterile PBS by oropharyngeal aspiration. Influenza A/PR/8/34 H1N1 was used to inoculate mice with 2000 PFU of influenza (in 50 μL sterile PBS) by oropharyngeal aspiration (provided as a gift by Radha Gopal, UPMC Children's Hospital of Pittsburgh, Pittsburgh, PA) (Constantinesco et al., 2024). This dose of influenza results in 15%–20% weight loss by day‐6 post‐influenza infection to mimic severe influenza infection. Viral burden was determined by quantitative real‐time RT‐PCR on lung RNA for viral matrix (M) protein. The gene expression levels of influenza M protein were analyzed by using the following primers: forward:5′‐GGACTGCAGCGTAGACGCTT‐3′, reverse:5′‐CATCCTGTTGTATATGAGGCCCAT‐3′, probe: 5′/56FAM/CTCAGTTAT/ZEN/TCTGCTGGTGCACTTGCCA/3IABkFQ/−3′. Mice were sequentially challenged with influenza or vehicle (PBS) followed by infection on Day 6 with either MRSA or vehicle (PBS) for an additional 24 h as previously described (Kudva et al., 2011; Robinson et al., 2013, 2014, 2015, 2018).

### 2.3 Analysis of lung inflammation

At the indicated time points, mouse lungs were lavaged with 1 mL sterile PBS for bronchoalveolar lavage cell counts and total protein levels (Bradford protein assay, Thermo Scientific Catalog #23200). The cranial lobe of the right lung was homogenized in sterile PBS by mechanical grinding. The resulting lung homogenate was used for bacterial colony counting and cytokine analysis by Bio‐Plex Multiplex immunoassay (Bio‐Rad, Catalog # M60009RDPD). Middle and caudal lobes of the right lung were snap‐frozen and homogenized under liquid nitrogen for RNA extraction using an RNA isolation kit (Agilent Technologies, Catalog #400800). RNA analysis was performed by standard RT‐PCR using Assay‐on‐Demand TaqMan probes and primers (Thermo Fisher Scientific Catalog #4331182 IFNβ Mm00439546_s1, IFNγ Mm01168134_m1, Rsad2 Mm00491264_m1, Ifit3 Mm01704846_s1, Ifit2 Mm00492606_m1, Isg20 Mm00469585_m1, Isg15 Mm01705338_s1, and Stat3 Mm01219775_m1).

### 2.4 Protein quantification

Whole cell lysates were extracted from homogenized cranial lobe of the right lung. Equal amounts of protein (20 μg) were separated on Nupage 4%–12% BisTris gels (Novex) and transferred to a polyvinylidene difluoride membrane. Target proteins were detected by immunostaining with specific primary antibody Phospho‐Stat3 Rabbit mAb (Cell Signaling Technology (CST) 9145) or beta‐actin (CST 4970) followed by Anti‐rabbit IgG HRP‐linked (CST 7074) secondary antibody. The specific immunoreactive bands were detected by chemiluminescence (Femto Electrochemiluminescence Substrate (Invitrogen Catalog #34096)). Quantification of band intensity (pixel density) was performed using NIH Image J software and normalized to beta‐actin.

### 2.5 ELISA assays

The levels of interferon β (R&D Systems Catalog # DY8234‐05), interferon λ (R&D Systems Catalog # DY1789B), and RAGE (R&D Systems Catalog # DY1179) were measured using sandwich ELISA using Duoset kits.

### 2.6 Histology and analysis

Left lung lobes from mice were inflated with and preserved in 10% neutral‐buffered formalin solution. Tissue sections were stained with H&E, and images were collected using an automatic high‐resolution microscopic scanner by Histowiz Inc. QuPath (https://qupath.github.io), an open‐source image analysis software, was used to analyze whole slide images. QuPath has a machine learning algorithm that uses pixel classification to identify objects once a trained data set has been established. For this study, lung sections from vehicle and influenza‐infected mice were used to train QuPath version 0.3.2 to identify healthy parenchyma and inflamed/injured parenchyma, as well as other lung structures, like blood vessels and airways. This training was then applied to all lung sections to identify changes in healthy versus inflamed/repairing parenchyma. This protocol has been published previously (Antos et al., 2024). Lung sections were also scored by blinded histopathologic evaluation, using a severity scale of 1 to 4, with 4 being the greatest amount of inflammation/injury as published previously (Sharma et al., 2022).

### 2.7 Statistics

All of the data are presented as the mean ± SEM except for continuous data which are presented as mean ± SD. Significance was tested by the unpaired *t*‐test (for 2 means) or 1‐way ANOVA after normality was confirmed. Mann–Whitney unpaired *t*‐test (for 2 means) or Kruskal–Wallis test with Dunn's multiple comparison test (for multiple data groups) was performed if normality test did not pass with alpha 0.05. All studies were repeated 2 times and collected data were combined for the figures unless otherwise described. Any statistical outlier was identified using Grubb's test and removed. Data were analyzed using GraphPad Prism software. *p* < 0.05 was considered significant.

### 2.8 Study approval

Mouse experiments were conducted with approval from the University of Pittsburgh Institutional Animal Care and Use Committee.

## 3 RESULTS

### 3.1 Baricitinib inhibits the JAK/STAT pathway in mouse models of post‐influenza staphylococcal pneumonia

**FIGURE 1:**
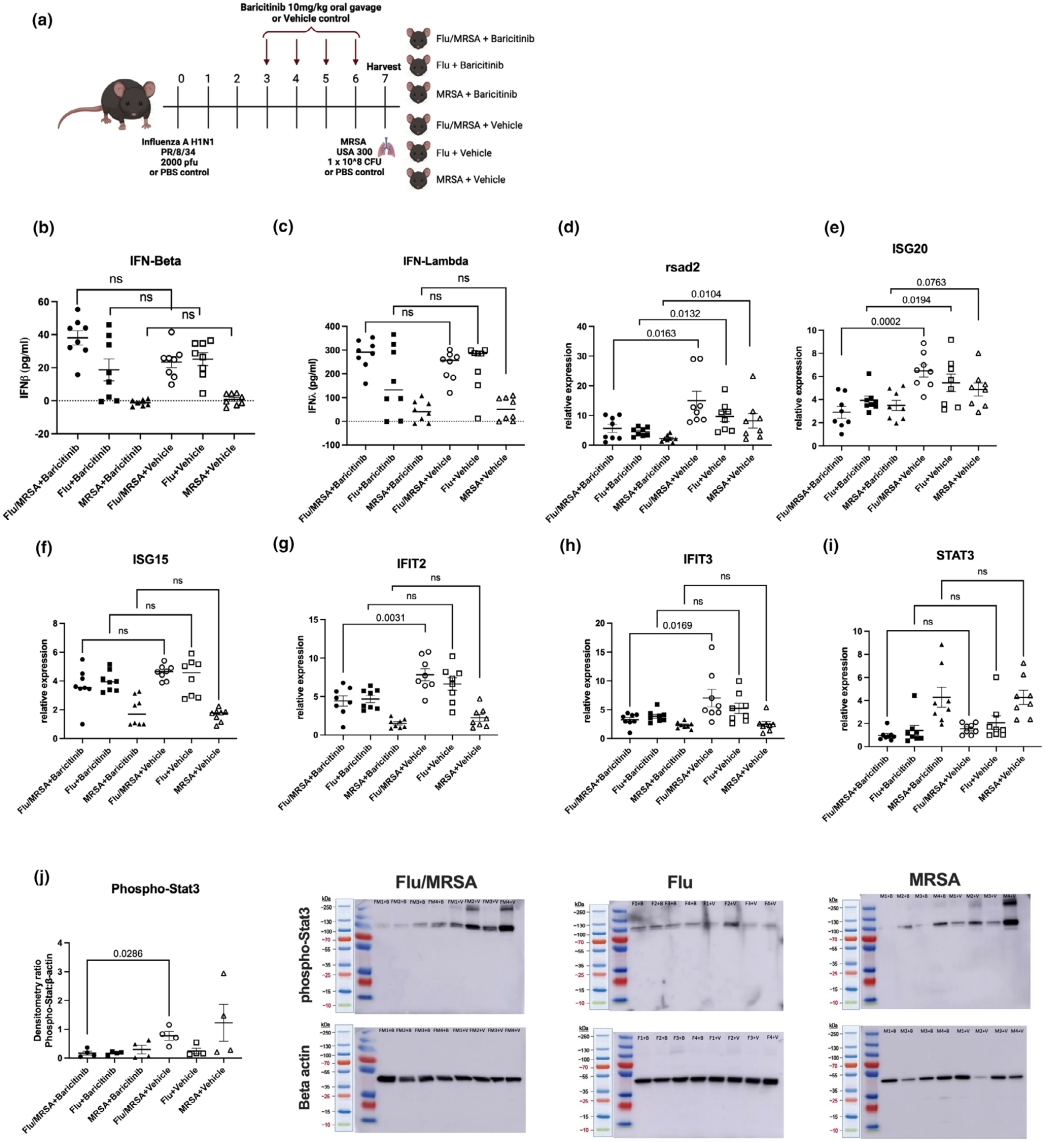
Baricitinib inhibits the JAK/STAT pathway in mouse models of post‐influenza staphylococcal pneumonia. (a) Experimental setup for post‐influenza MRSA pneumonia. C57BL/6 mice were infected with 2000 pfu of Influenza A/PR/8/34 or vehicle for 6 days, mice were then challenged with 1 × 10^8^ CFU of MRSA USA 300 for 24 h. Mice received either baricitinib or vehicle control on days 3–6 post‐influenza infection. (b, c) Expression levels of interferons beta and lambda using ELISA. (d–i) Gene expression of interferon stimulating genes and STAT3 in lung RNA (*n* = 8). (j) Western Blot and densitometry for phosphorylated STAT3 (*n* = 4). Data points reflect individual values ± SEM. Each experiment was independently performed twice with the exception of panel j, which was performed once, and data are shown from combined experiments. Significance was tested by the unpaired *t*‐test (for 2 means) or 1‐way ANOVA after normality was confirmed. Mann–Whitney unpaired *t*‐test (for 2 means) or Kruskal–Wallis test with Dunn's multiple comparison test (for multiple data groups) was performed if normality test did not pass with an alpha of 0.05.

Given its proven effectiveness in mouse models (Gu et al., 2023; Matsushita et al., 2023) and clinical benefit during SARS‐CoV‐2 infection, we treated mice with the FDA‐approved JAK inhibitor baricitinib during influenza infection, staphylococcal pneumonia, and post‐influenza MRSA pneumonia (Figure 1a) (Ely et al., 2022; Group RC, 2022; Marconi et al., 2021; Wolfe et al., 2022). Janus kinase (JAK) mediates the downstream effects of interferons by increasing the expression of interferon‐stimulated genes (ISGs). To confirm that baricitinib inhibited the JAK pathway in our mouse model, we examined the expression of interferons and ISGs in mice infected with influenza, MRSA, or post‐influenza MRSA. Our data show that, compared to MRSA infection, influenza infection strongly induced both type I (Figure 1b) and type III interferons (Figure 1c). Post‐viral bacterial infections did not further increase the expression of interferons compared to influenza infection alone. As expected, given its downstream sites of action, baricitinib treatment did not reduce the expression of Type I or Type III interferons. However, baricitinib significantly attenuated the expression of ISGs rsad2 and ISG20 during influenza infection, MRSA pneumonia, and post‐influenza MRSA pneumonia. This effect was observed across multiple ISGs, including rsad2, ISG20, IFIT2, and IFIT3 during post‐influenza MRSA pneumonia (Figure 1d–h). These findings suggest that baricitinib effectively inhibits the JAK pathway in the lungs in our mouse model. Furthermore, our data also show that STAT3 activation was most pronounced in MRSA infection compared to viral/bacterial super‐infection; however, there were no differences between the baricitinib and vehicle treatment groups (Figure 1i). During post‐influenza MRSA pneumonia, baricitinib significantly reduced levels of phosphorylated STAT3 (Figure 1j). Together, these results demonstrate that oral baricitinib effectively inhibits the JAK/STAT pathway in mouse models of influenza infection and post‐influenza MRSA pneumonia.

### 3.2 Baricitinib inhibits select cytokine responses during influenza infection, staphylococcal pneumonia, and post‐influenza MRSA pneumonia

**FIGURE 2:**
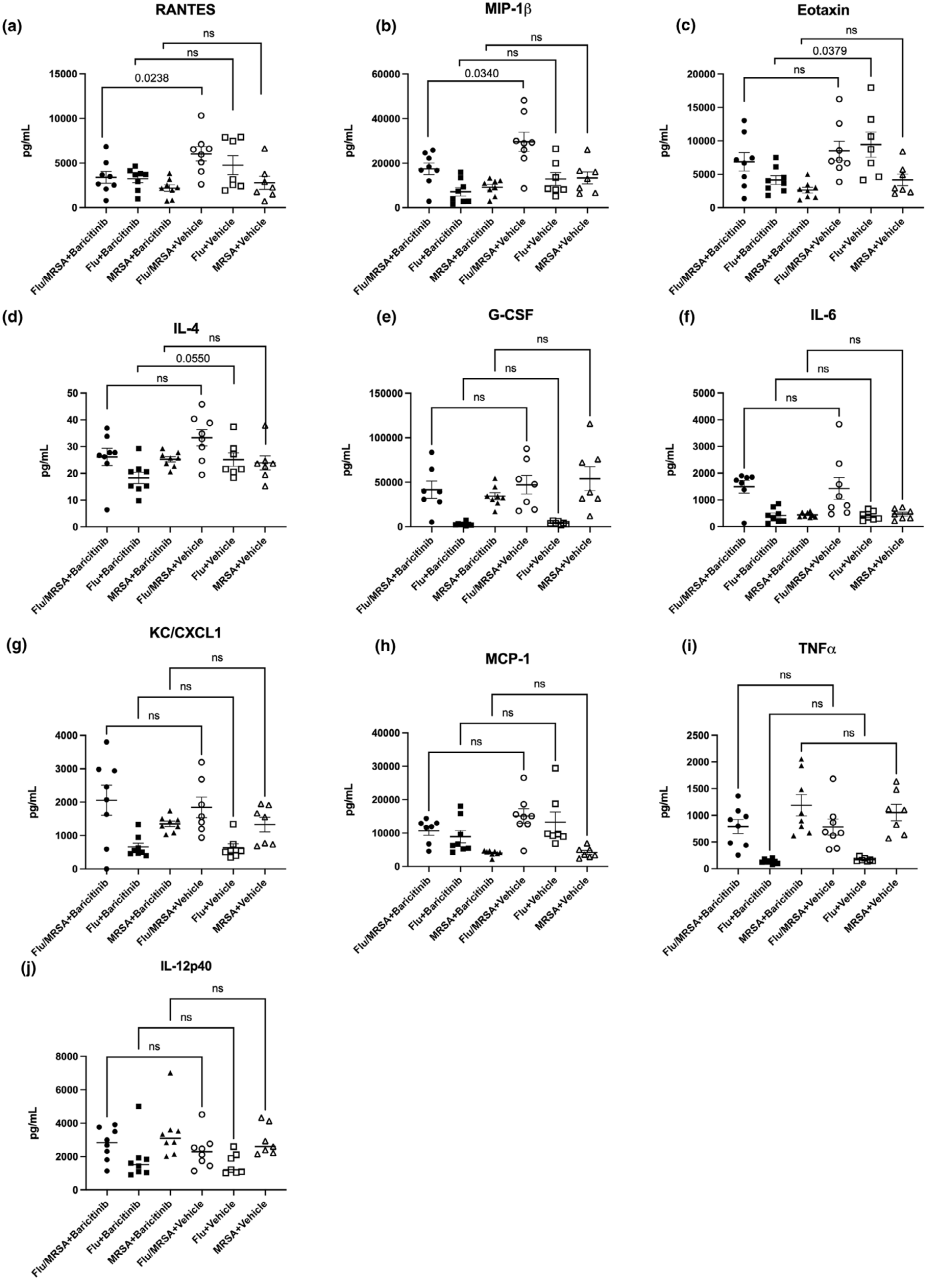
Baricitinib inhibits specific cytokine responses during influenza infection, Staphylococcal pneumonia, and post‐influenza MRSA pneumonia. (a–j) Cytokine and chemokine protein concentrations in lung homogenate measured by mouse 23‐plex immunoassay (*n* = 8). Data points reflect individual values ± SEM. Each experiment was independently performed twice, and data are shown from combined experiments. Significance was tested by the unpaired *t*‐test (for 2 means) or 1‐way ANOVA after normality was confirmed. Mann Whitney unpaired *t*‐test (for 2 means) or Kruskal–Wallis test with Dunn's multiple comparison test (for multiple data groups) was performed if normality test did not pass with alpha 0.05.

To determine how baricitinib inhibition of the JAK/STAT pathway affects inflammation in the lungs during influenza infection, staphylococcal pneumonia, and post‐influenza MRSA pneumonia, we measured inflammatory cytokines in lung homogenates. Our data show that JAK inhibition did not lead to a global reduction in cytokine secretion in the lungs. Instead, we observed selective modulation of specific cytokines during post‐influenza MRSA pneumonia including RANTES and MIP‐1β (Figure 2a,b). Additionally, we observed decreased levels of eotaxin and IL‐4 during influenza infection alone in mice treated with baricitinib (Figure 2c,d). No differences were observed in G‐CSF, IL‐6, KC, MCP‐1, TNFa, and IL‐12p40 (Figure 2e–j). These data indicate that baricitinib largely does not impact the host inflammatory response during influenza infection, staphylococcal pneumonia, and post‐influenza MRSA pneumonia despite JAK pathway inhibition.

### 3.3 Baricitinib treatment does not impair host immunity or exacerbate tissue injury during influenza infection, staphylococcal pneumonia, and post‐influenza MRSA pneumonia

**FIGURE 3:**
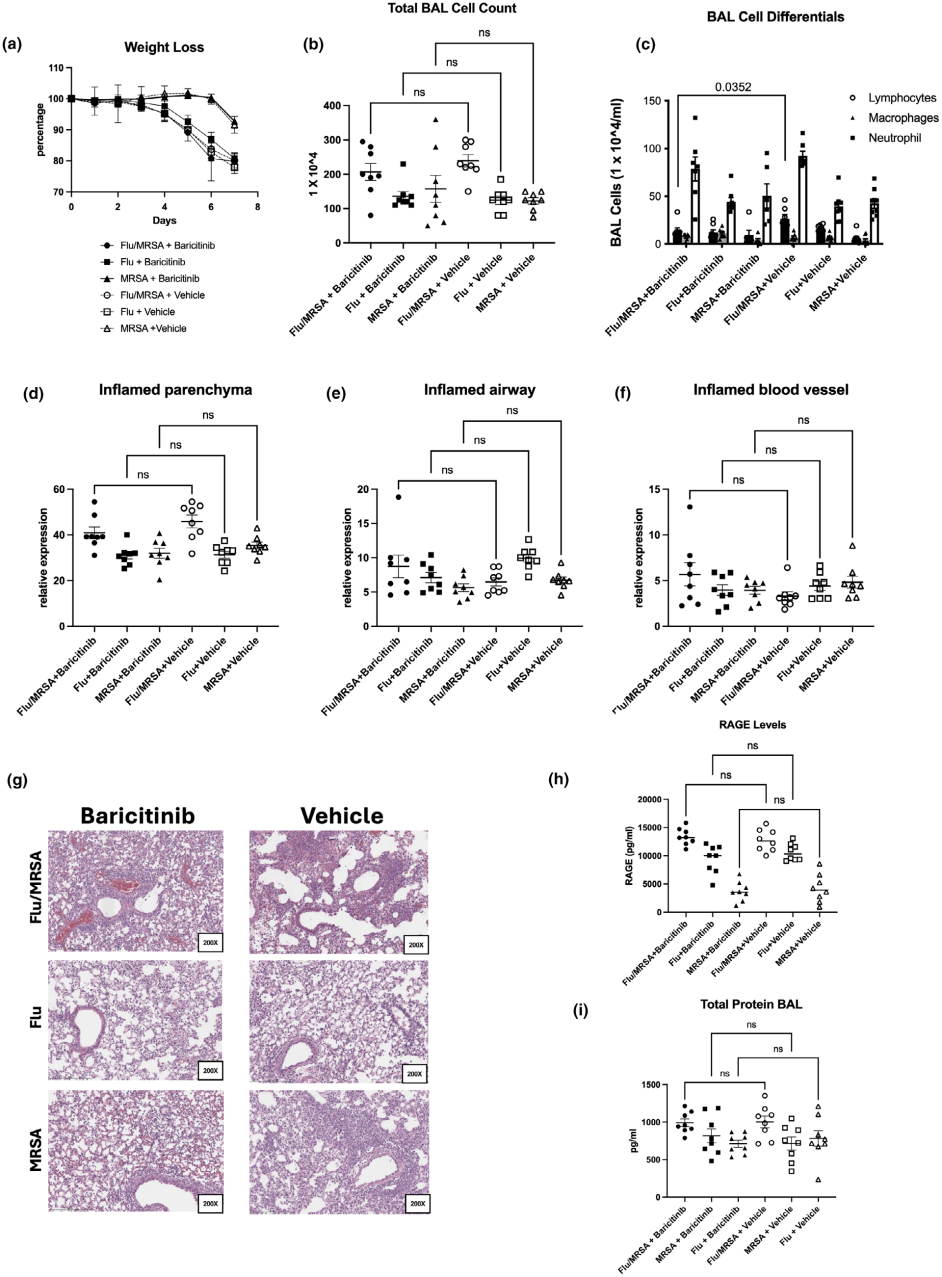
Baricitinib treatment does not exacerbate tissue injury during influenza infection, Staphylococcal pneumonia, and post‐influenza MRSA pneumonia. (a) Weight loss measured throughout the course of infection (*n* = 8). (b) BAL fluid cell counts (*n* = 8). (c) BAL fluid cell differential counts for lymphocytes, macrophages, and neutrophils. (d–g) Scoring of inflamed parenchyma, airway, and blood vessels (*n* = 8) and representative histology of H&E‐stained left lung sections. (h) Levels of RAGE in the BAL as a marker of epithelial cell death, and (i) total protein in BAL fluid as a marker of vascular permeability (*n* = 8). (a) Data points reflect individual values ± SD. (b–i) Data points reflect individual values ± SEM. Each experiment was independently performed twice, and data are shown from combined experiments. Significance was tested by the unpaired *t*‐test (for 2 means) or 1‐way ANOVA after normality was confirmed. Mann–Whitney unpaired *t*‐test (for 2 means) or Kruskal‐Wallis test with Dunn's multiple comparison test (for multiple data groups) was performed if normality test did not pass with alpha 0.05.

**FIGURE 4:**
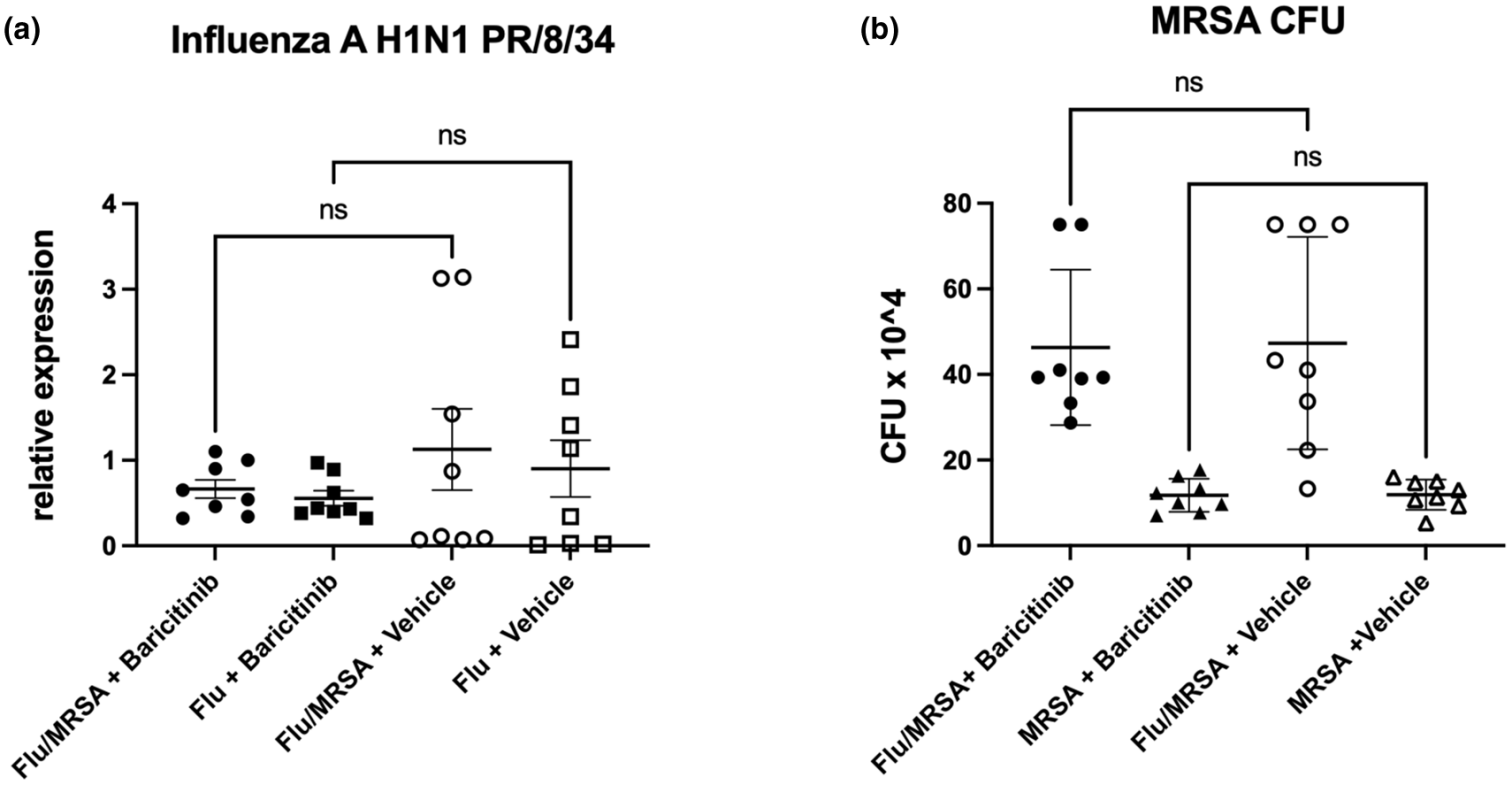
Baricitinib treatment does not impair host immunity during influenza infection, Staphylococcal pneumonia, and post‐influenza MRSA pneumonia. (a) Influenza matrix protein expression in lung RNA (*n* = 8). (b) Bacterial colony counts in lung homogenate (*n* = 8). Data points reflect individual values ± SEM. Each experiment was independently performed twice, and data are shown from combined experiments. Significance was tested by the unpaired *t*‐test (for 2 means) or 1‐way ANOVA after normality was confirmed. Mann–Whitney unpaired *t*‐test (for 2 means) or Kruskal–Wallis test with Dunn's multiple comparison test (for multiple data groups) was performed if normality test did not pass with alpha 0.05.

To understand the effects of baricitinib treatment on infection outcomes, we investigated its impact on host defense and tissue injury. As expected, influenza infection caused significant body weight loss compared to MRSA infection alone (Figure 3a). Baricitinib treatment did not alter weight loss trajectories in our mouse models, including influenza infection, staphylococcal pneumonia, and post‐influenza MRSA pneumonia (Figure 3a). Consistent with the cytokine findings above, baricitinib did not impact the cellular inflammatory response in the lungs, as evidenced by inflammatory infiltration in the bronchoalveolar lavage (BAL) fluid. The total BAL cell count was increased in post‐influenza MRSA infection compared to singular infections, with no alteration in post‐influenza MRSA mice that received baricitinib treatment. Although there was a trend towards decreased BAL cell count in the singular infections that received baricitinib treatment compared to the post‐influenza MRSA mice that received baricitinib treatment, there was no statistical difference between any of the baricitinib groups (Figure 3b). Furthermore, there were no differences observed in neutrophil or macrophage counts in the BAL between baricitinib‐treated mice or vehicle‐treated mice. However, there was a decrease in total lymphocytes in the post‐influenza MRSA infection that received baricitinib compared to vehicle (Figure 3c). Lung tissue showed increased areas of inflamed parenchyma in the post‐influenza MRSA group compared to singular infections, with no differences between the baricitinib and vehicle groups (Figure 3d–g). Epithelial injury was assessed by measuring the levels of RAGE in the BAL samples, which was increased in the super infection group compared to individual infections. However, we did not observe any differences between vehicle and baricitinib groups (Figure 3h). Similarly, vascular leak, measured as total protein content in the BAL, was unchanged between all treatment groups (Figure 3i). Finally, we assessed whether baricitinib treatment affected the host's ability to clear infecting pathogens in our models. Baricitinib treatment did not alter influenza viral load (Figure 4a) or MRSA bacterial load (Figure 4b). As previously reported (Robinson et al., 2013, 2014, 2015, 2018), influenza impaired the host's ability to clear MRSA infection during post‐influenza MRSA pneumonia (Figure 4b). Together, these data suggest that baricitinib treatment during influenza infection does not alter viral burden or impair host defense against secondary bacterial infections in the lung.

### 3.4 Interleukin‐6 deficiency does not affect host defense against post‐influenza MRSA pneumonia

**FIGURE 5:**
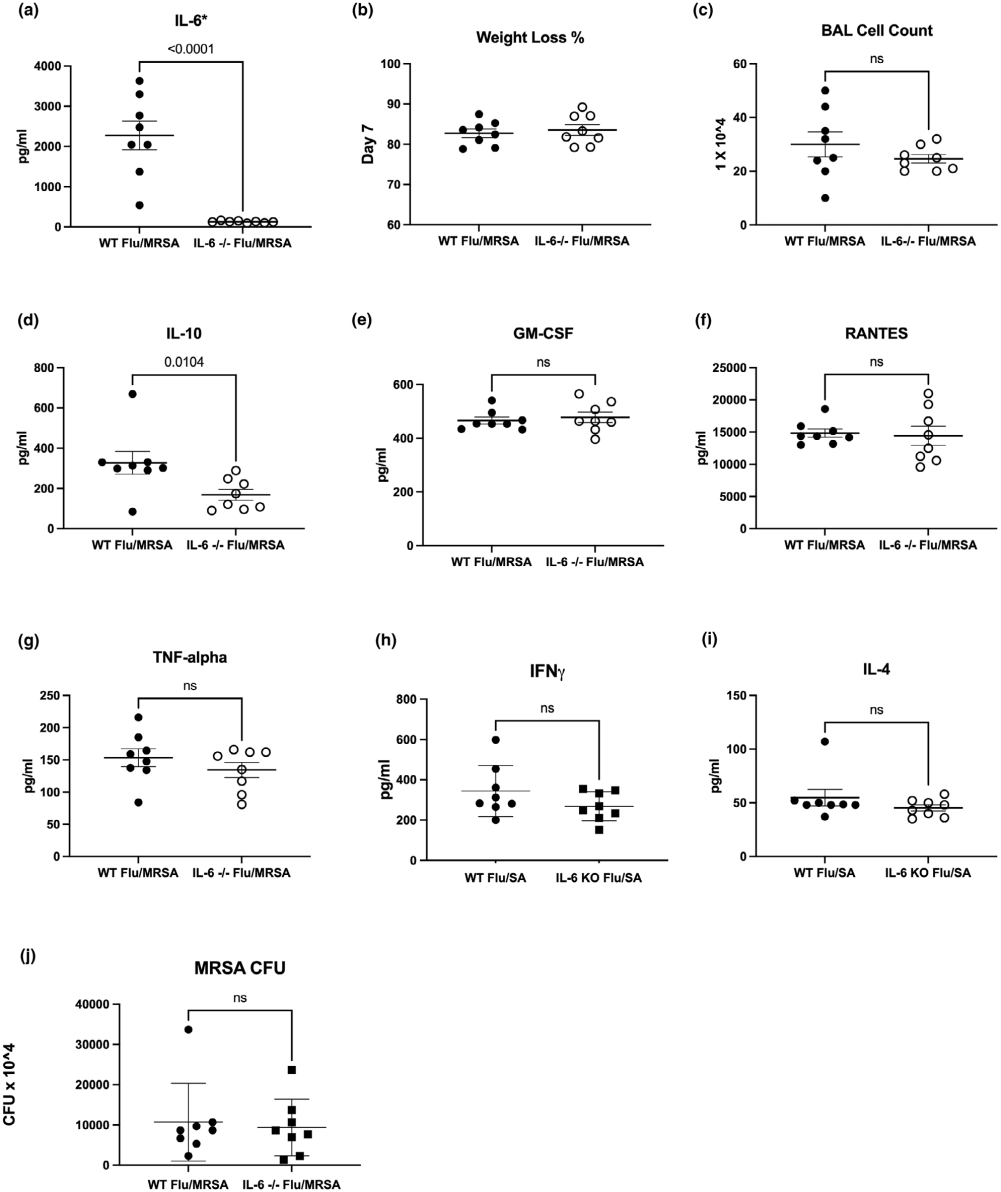
Interleukin‐6 deficiency does not affect host defense against post‐influenza MRSA pneumonia. C57BL/6 mice (wild‐type) and IL‐6−/− mice were infected with 2000 pfu of Influenza A/PR/8/34 or vehicle for 6 days, mice were then challenged with 5 × 10^7^ CFU of MRSA USA 300 for 24 h. (a) IL‐6 concentrations in lung homogenate measured by mouse 23‐plex immunoassay (*n* = 8). (b) Weight loss measured on Day 7 post‐infection (*n* = 8). (c) BAL fluid cell counts (*n* = 8). (d–h) Cytokine, chemokine, and interferon γ concentrations in lung homogenate measured by mouse 23‐plex immunoassay (*n* = 8). (j) Bacterial colony counts in lung homogenate (*n* = 8). Data points reflect individual values ± SEM. Each experiment was performed once. Significance was tested by the unpaired *t*‐test (for 2 means) or 1‐way ANOVA after normality was confirmed. Mann–Whitney unpaired *t*‐test (for 2 means) or Kruskal–Wallis test with Dunn's multiple comparison test (for multiple data groups) was performed if normality test did not pass with alpha 0.05.

Given that JAK inhibition had the greatest effect on post‐influenza MRSA pneumonia, and IL‐6 activates the JAK/STAT pathway, we also investigated how deficiency of IL‐6 signaling might affect host susceptibility to post‐influenza bacterial pneumonia. IL‐6 receptor antagonists such as tocilizumab are ineffective in mice (Lokau, Kleinegger, et al., 2020; Lokau, Waetzig, et al., 2020; Okazaki et al., 2002), so we utilized IL‐6 knockout mice to explore how IL‐6 impairment affects post‐influenza MRSA pneumonia. First, we confirmed the absence of IL‐6 protein in our knockout mice (Figure 5a). We found that IL‐6 deficiency does not alter weight loss at day 7 post‐influenza MRSA pneumonia compared to wild‐type (WT) mice (Figure 5b). Additionally, inflammatory cell recruitment in the lungs was similar regardless of the presence of IL‐6 (Figure 5c). IL‐6 KO mice have a similar inflammatory cytokine profile to WT mice during post‐influenza MRSA pneumonia, including GM‐CSF, RANTES, TNFα, IFNγ, and IL‐4. However, we observed a significant decrease in IL‐10 levels in the absence of IL‐6 (Figure 5d–i). The absence of IL‐6 did not change bacterial burden in the lung during post‐influenza MRSA pneumonia (Figure 5j). Together, these data suggest that the absence of IL‐6 does not impair host defense against post‐influenza bacterial super‐infection in the lung.

## 4 DISCUSSION

In this study, we explored the impact of immunomodulatory therapies on host defense during influenza infection. We focused on IL‐6 and JAK signaling using baricitinib and IL‐6‐deficient mouse models, based on beneficial effects demonstrated during SARS‐CoV‐2 infection. Given the high rate of secondary bacterial pneumonia during the 2009 influenza pandemic and proposed use of immunomodulators during severe influenza infection, we sought to determine if JAK inhibition altered host immunity to singular influenza infection, singular bacterial infection, or post‐influenza bacterial super‐infection.

IL‐6 is an immunomodulatory cytokine with both pro‐ and anti‐inflammatory effects that regulates numerous disease processes. In classic IL‐6 signaling, IL‐6 binds to the membrane bound IL‐6 receptor to activate signal‐transducing glycoprotein 130 while in IL‐6 trans‐signaling, soluble forms of the IL‐6 receptor form complexes with IL‐6 to activate membrane bound glycoprotein 130. Receptor dimerization activates JAKs which phosphorylate themselves and the receptor and serves as a docking site for STATs. Phosphorylated STATs translocate to the nucleus and induce gene transcription. The JAK/STAT pathway mediates the effects of cytokines and interferons. Interferons are highly upregulated during influenza infection. Both classic and trans‐signaling of IL‐6 can be inhibited by antibodies and small molecules directed at IL‐6, the IL‐6 receptor, or downstream JAKs (Rose‐John et al., 2023). Baricitinib is a JAK inhibitor used to treat SARS‐CoV‐2 infection, and it has been hypothesized that it may also prove useful as a therapeutic agent during influenza infection. Thus, we utilized baricitinib in mouse models of influenza infection, salterntaphylococcal pneumonia, and post‐influenza staphylococcal pneumonia to determine its effects. Daily baricitinib has been approved for use in humans with COVID‐19 infection who are hospitalized and requiring supplemental oxygen, mechanical ventilation, or extracorporeal membrane oxygenation for a total of 14 days or until hospital discharge. In our studies, mice were treated with baricitinib on day 3 through day 6 post‐influenza infection with tissues harvested on Day 7 post‐influenza infection, to mimic severe influenza infection when patients have become ill and presented to the hospital. Notably, humans receive 4 mg daily of baricitinib, while in our mouse studies we used 10 mg/kg daily, using a dose proven to be effective in prior mouse studies (Gu et al., 2023; Matsushita et al., 2023; Tuttle et al., 2020).

Our results demonstrate that baricitinib effectively inhibits the JAK/STAT pathway in mouse models of influenza infection and post‐influenza MRSA pneumonia. This is evidenced by the significant reduction in interferon‐stimulated genes (ISGs) without altering interferon expression and a decrease in phosphorylated STAT3 levels. Interestingly, our data shows that baricitinib does not improve viral clearance or decrease inflammatory tissue injury during singular influenza infection, nor does it exacerbate susceptibility to secondary bacterial pneumonia in mice. These conclusions are supported by the lack of significant differences in weight loss, lung pathology, vascular leak, and pathogen clearance between baricitinib‐treated and vehicle‐treated groups during singular influenza infection and post‐influenza MRSA pneumonia. Importantly, both viral burden and bacterial burden are unchanged between the baricitinib‐treated and vehicle‐treated groups. Baricitinib treatment selectively modulated certain inflammatory cytokines while largely leaving the overall inflammatory response unaffected. Overall, these results suggest that baricitinib may not improve tissue injury or pathogen clearance if used during influenza infection or post‐influenza MRSA pneumonia.

Prior studies have investigated the use of baricitinib during both influenza infection and following Poly(I:C) administration in mice (Tuttle et al., 2020; Yu et al., 2024). Poly (I:C) is a synthetic analog of double‐stranded RNA, a molecular pattern associated with viral infections. Similar to our findings, when baricitinib was started on day‐3 post‐influenza infection, Yu et al. observed no differences in weight loss in mice that received baricitinib compared to placebo control (Yu et al., 2024). The experiments described did not investigate the host immune response; however, they observed a reduction in pro‐inflammatory cytokines and chemokines with JAK inhibition, but no change in viral burden compared to placebo control (Yu et al., 2024). When baricitinib was given during acute Poly(I:C) systemic administration by Tuttle et al., an impact on early cytokine production was observed at 4 h post‐challenge, but these changes were not sustained (Tuttle et al., 2020). Our current studies seem to align with this previously published literature (Tuttle et al., 2020; Yu et al., 2024). Although other JAK inhibitors may warrant additional investigation, baricitinib does not improve pathogen clearance or inflammatory tissue damage in our murine models of influenza or post‐influenza MRSA pneumonia.

IL‐6 is highly upregulated following influenza infection and has shown to be protective in animal models with IL‐6 deficient mice having increased mortality and lung damage compared to wild‐type controls (Dienz et al., 2012; Yang et al., 2017). During post‐influenza streptococcal pneumonia, IL‐6 deficient mice have increased bacterial burden and mortality compared to control mice (Gou et al., 2020). Monoclonal antibodies targeting the IL‐6 receptor or cytokine‐receptor binding affinity are used in SARS‐CoV‐2 infection and have shown to improve survival (REMAP‐CAP Investigators et al., 2021). Given the poor survival observed in IL‐6 deficient mice during influenza and post‐influenza streptococcal pneumonia and the hypothetical use of IL‐6 antagonists during influenza infection, we sought to determine the role of IL‐6 inhibition during post‐influenza MRSA pneumonia. Tocilizumab (Lokau, Kleinegger, et al., 2020; Lokau, Waetzig, et al., 2020; Okazaki et al., 2002) has previously been shown to be ineffective in mouse models, so we elected to use IL‐6‐deficient mice to model IL‐6 inhibition during post‐influenza MRSA pneumonia. We observed that IL‐6 deficiency did not impair the host's ability to defend against post‐influenza MRSA pneumonia. IL‐6 knockout mice displayed similar weight loss trajectories, inflammatory cell recruitment, and pathogen clearance as wild‐type mice. Although there was a significant reduction in IL‐10 levels in IL‐6‐deficient mice, this did not alter susceptibility to secondary bacterial infection. Notably, mice lacking IL‐10 have improved clearance of MRSA during post‐influenza bacterial pneumonia (Robinson et al., 2015). Interestingly, IL‐6 deletion did not exacerbate post‐influenza MRSA pneumonia like it has done in prior published studies of singular influenza infection or post‐influenza streptococcal pneumonia. However, IL‐6 deletion also did not lessen bacterial burden compared to wild‐type controls. IL‐6 deficient mice are known to have decreased IFNγ secretion, increased IL‐4 secretion, and decreased pro‐B cell numbers in the bone marrow compared to wild‐type mice (https://www.jax.org/strain/002650). Notably, we did not observe any differences in IFNγ or IL‐4 between wild‐type and IL‐6 deficient mice in our model. Future studies are warranted to determine how IL‐6 inhibition, versus total deletion, may affect influenza infection or post‐influenza bacterial pneumonia. IL‐6 may be protective during influenza infection; however, excessive levels of IL‐6 may cause cytokine storm and lung injury. IL‐6 inhibition may have different effects if used during post‐influenza bacterial pneumonia caused by different types of bacteria or if IL‐6 is inhibited at different time points during the course of infection.

Our study suggests that IL‐6 and JAK inhibition during influenza infection may not lead to improved pathogen clearance or limit tissue injury, in contrast to what has been observed in SARS‐CoV‐2 infection in humans. These differences may be attributed to different pathogenesis of these diseases, where COVID‐19 is associated with higher systemic disease (Xiao et al., 2024) compared to influenza. Further studies are warranted to understand the effect of JAK inhibition in other common respiratory infections such as those caused by RSV, rhinovirus, and adenovirus. While our findings in mouse models provide valuable insights, limitations of our studies include inherent differences in immune responses between species and that differences in timing and dosage of immunomodulatory therapy may provide different results. Importantly, we initiated baricitinib therapy on day 3 post‐influenza infection to provide a clinically relevant murine translational study and have demonstrated that baricitinib effectively inhibits JAK/STAT signaling and ISG induction in our model. Further, our studies included a 10 mg/kg dose as indicated in prior publications, which is much higher than clinically used human dose of 4 mg per day (Bronte et al., 2020). However, this dose is required in mouse models to achieve JAK inhibition as shown in our study and prior studies. Another limitation of this model is our investigation being focused on lung pathology, which is due to localized tissue injury in mouse models of influenza. However, clinical manifestations of severe disease such as influenza or post‐influenza MRSA have systemic aspects of the disease. These findings support careful consideration of immunomodulatory therapies in managing severe influenza infection and highlight the need for additional research in this area.

## FUNDING INFORMATION

This work is supported by funding from NIH to KMR (1R01AI153337) and the United States Veteran's Affairs to WB (IK2BX004886).

## ETHICS STATEMENT

All mouse experiments were approved by the University of Pittsburgh IACUC, Protocol #: 24064977.

## Data Availability

All the raw data are available from the corresponding author upon reasonable request.
